# Reliability, Knowledge Translation, and Implementability of the Spanish Version of the Hammersmith Infant Neurological Examination

**DOI:** 10.3390/healthcare12030380

**Published:** 2024-02-01

**Authors:** Álvaro Hidalgo-Robles, Javier Merino-Andrés, Ángel Luis Rodríguez-Fernández, Mónica Gutiérrez-Ortega, Irene León-Estrada, Maribel Ródenas-Martínez

**Affiliations:** 1 Facultad de Educación, Universidad Internacional de La Rioja, 26006 Logroño, Spain; alvaro.hidalgo@unir.net (Á.H.-R.); irene.leon@unir.net (I.L.-E.); 2 Faculty of Physiotherapy and Nursing, Universidad de Castilla-La Mancha, 45004 Toledo, Spain; 3 Physiotherapy Research Group of Toledo (GIFTO), Universidad de Castilla-La Mancha, 45004 Toledo, Spain; 4 Facultad de Medicina, Universidad CEU San Pablo, 28668 Alcorcón, Spain; alrodfer@ceu.es; 5 Facultad de Educación y Trabajo Social, Universidad de Valladolid, 47011 Valladolid, Spain; monica.gutierrez.ortega@uva.es; 6 Asociación APSA, 03005 Alicante, Spain; maribelrm@asociacionapsa.com

**Keywords:** Hammersmith infant neurological examination, early detection, cerebral palsy, reliability, knowledge translation

## Abstract

*Purpose*. This study aimed to: (a) translate and cross-culturally adapt the Hammersmith Infant Neurological Examination (HINE) into Spanish; (b) evaluate its intra- and inter-examiner reliability; (c) support a knowledge translation and tool implementation program in early intervention; and (d) evaluate its reliability and implementation for professionals one year after receiving training. *Materials and methods*. The translation followed the World Health Organization’s recommendations. Reliability was assessed in 25 infants aged between 3 and 15 months with identifiable risks of cerebral palsy (CP). The implementation was also evaluated by analyzing the reliability of professionals without previous experience of the tool by using a pre-survey and a follow-up survey one year after training. The survey covered aspects related to the use of early detection tools of CP and the use of HINE, including attitudes, opinions, and perceptions. *Results*. An excellent intra- and inter-examiner agreement was obtained for the total score of the HINE intra-class correlation coefficient (ICC = 0.98 in both indices). One year after training, the professionals also showed excellent reliability values (ICC = 0.99), as well as an increase in sensitization and skills in evidence-based practices for the early detection of “high risk” of CP. *Conclusions*. The Spanish version of HINE is a reliable measure for the neurological evaluation of “high risk” of CP and can be administered after standardized training and without costs to acquire the evaluation. This allows its accessible and widespread implementation in the clinical context.

## 1. Introduction

Cerebral palsy (CP) is one of the most common movement and posture disorders, with a prevalence of 1.6/1000 live births, although with a significant decreasing trend for pre- and peri-natal CP in Europe and Australia [1]. Despite the lack of epidemiological registers in Spain [2], it is estimated that there are significant differences between Spanish regions attributed to a disparity in diagnostic practices, especially at an early age [3]. This may lead to a delayed follow-up of the “high-risk population for CP” and late referral to early intervention services [4,5].

Compared with the option of conservative follow-up, the scientific literature has shown that CP or “high risk for CP” can be diagnosed accurately and early—often under 6 months of age—using clinical reasoning and a combination of standardized measures [4,6]. Within these measures, neurological evaluation plays an essential role in the follow-up programs for the high-risk population, supporting the diagnosis and defining the prognosis, monitoring the longitudinal history of the condition, and documenting the effects of the interventions [7].

The Hammersmith Infant Neurological Examination (HINE) is a simple, quantifiable, easy-to-use, and widely studied neurological evaluation used in typically developing and high-risk children between 2 and 24 months of age [8]. It was developed in 1999 by Haataja et al. and is composed of three sections: neurological evaluation, motor development, and behavior. The total HINE score is based solely on the first section, which includes 26 items and examines neurological function (cranial nerve function, posture, movements, tone and reflexes, and reactions). Together with Prechtl’s General Movement Assessment and neuroimaging, it is recommended by international clinical practice guidelines, attaining a positive predictive value of 90% [4,9]. With the existence of an optimal score range [10] and the possibility of a quantitative assessment of neurological findings and developmental monitoring, HINE can be used for clinical and research purposes.

The development of brief training workshops that include theoretical, research, and practical aspects of HINE has proven effective for the subsequent implementation of the tool in the United States [7]. Cross-cultural adaptations and studies of the psychometric properties of HINE in Indian and Turkish populations provide excellent intra- and inter-examiner correlation coefficients and contribute to knowledge translation [11,12]. Similarly, studies investigating intra-examiner reliability in the United States [13] and inter-examiner reliability in Italy [14] and Norway [15] consistently support the applicability of HINE across diverse cultural contexts. In the absence of more data about the psychometric properties that allow comparative studies of international scope —also for those professionals who have participated in a standardized HINE training program—the cross-cultural adaptation of HINE to the Spanish population is essential. Following the World Health Organization’s recommendations on the development and use of standardized health indicators [16], the objectives of this study include: (a) obtaining a cross-cultural translation and adaptation of HINE; (b) assessing HINE’s intra- and inter-examiner reliability in professionals experienced with the tool; (c) supporting knowledge translation processes through the implementation of training programs and the adaptation of different materials; and (d) evaluating the use of early detection tools of CP, including HINE and its inter-examination reliability, and attitudes, opinions, and perceptions of professionals one year after receiving training.

## 2. Materials and Methods

### 2.1. Description of HINE

HINE comprises 37 items divided into 3 sections: neurological evaluation, motor development, and behavior [10]. The 1st section of HINE examines neurological functions and includes 26 items. The 2nd section assesses motor development through 8 items. The 3rd section assesses behavior and includes 3 items. In the neurological assessment section only, each item is scored individually—from 0 to 3—according to descriptions and illustrative diagrams. The total score is the sum of all individual scores and can range from 0 to 78. The second and third sections are not part of the total score but provide important additional information regarding progress of development. Similarly, if asymmetries appear in the child, this must be recorded when presenting the items, with a score of 1 if they exist and 0 if not.

The application time of HINE varies between 5 and 15 min [8,17]. The examination is carried out through the intervention of the evaluator, who follows the instructions for each item as requested in the score sheet. This allows obtaining the responses concerning the child to determine the score and the asymmetries according to the child’s degree of response.

The cross-cultural adaptation of HINE consists of three phases: (i) translation and cross-cultural adaptation into Spanish from Spain; (ii) adaptation of a training program, guidelines of recommendations, and adapted materials; (iii) intra- and inter-examiner reliability analysis; (iv) analysis of intra- and inter-examiner reliability of professionals one year after training.

### 2.2. Cross-Cultural Translation and Adaptation

The HINE score sheet was translated into Spanish from the original language—English—and back-translated following the recommendations of the World Health Organization and publications and expert opinions for the translation, adaptation, and interpretation of protocols [16,18,19].

### 2.3. Participants

This study targets children at risk of developing CP, identified as a population with identifiable risk at birth or with identifiable risk in the child aged between 3 and 15 months and residing in Spain. Participants were recruited from early intervention services in the provinces of Valencia, Alicante, and Toledo (Spain). In line with the existing scientific literature, the inclusion criteria encompassed identifiable risk characteristics at birth: prematurity, neonatal encephalopathies, history of neurological risk factors, e.g., newborns with congenital disabilities, intrauterine growth retardation, and/or being admitted to neonatal intensive care units. Similarly, other identifiable factors in the child, such as identifying concerns in the family, the inability to sit at 9 months, or manual asymmetry, were considered [4].

Systemic diseases, orthopedic deformities, or medications that could affect alertness during the evaluation process were considered exclusion criteria. This study was approved by the Research Ethics Committee of the Universitat Internacional de Catalunya (FIS-2019-01).

### 2.4. Evaluation of Intra- and Inter-Examiner Reliability

The assessment of intra- and inter-examiner reliability involved two steps: (i) a preliminary and preparatory study; (ii) intra- and inter-examiner reliability analysis.

The preliminary study involved 25 participants aged between 3 and 12 months. Three physiotherapists with experience and training in pediatrics (AH-R, JM-A, and MR-M) administered HINE to the same patients after completing a 4 h training led by two HINE instructors who had published articles on the tool. The initial in-person HINE assessment was conducted by one physiotherapist (AH-R), and subsequent evaluations were based on the review of the video recording of the initial assessment. The preliminary results provided excellent intra-examiner reliability (intra-class correlation coefficient, ICC = 0.97) and good inter-examiner reliability (ICC = 0.67) for the total score. Following this phase, meetings were conducted with the evaluators to consolidate the application of common criteria during the evaluation and to develop recommendation guidelines based on the detailed analysis of the results obtained.

For the intra- and inter-examiner analysis, the assessment of 25 different participants was performed in person by one of the evaluators (MR-M) and video-recorded to allow the subsequent evaluations following the methodology used in the preliminary study and consistent with previous studies of the tool’s psychometric properties [11,12]. The video recordings were sent without any additional information to the two remaining evaluators (AH-R and JM-A), who analyzed them independently and without any communication between them to determine inter-examiner reliability. Through video analysis, a second evaluation was carried out by the first evaluator to perform a test–retest in a period of not less than 15 days, thus eliminating recall bias. In this way, we could evaluate the degree of reliability of HINE at the level of reproducibility and repeatability, estimating the intra-examiner reliability.

### 2.5. Training Program and Development of the Recommendation Guidelines for Evaluation with HINE in Spanish

In order to adapt an impactful training program based on successful international experiences and to evaluate its impact on the professional practice of Spanish clinicians, we trained professionals in the use and application of HINE items, structured by adapting the modules developed by Maitre et al. [7] (Table 1). To this program, we developed a document with recommendations for assessment with HINE in Spanish, following different materials published in studies on the tool and general recommendations by experts.

To ensure the reliability of the HINE training program, an additional study was conducted to evaluate the inter-examination reliability in two early intervention services in Valencia (Spain) one year after receiving HINE training. Additionally, pre- and post-training surveys were conducted in December 2020 and December 2021, respectively, with 18 questions related to the use of early detection tools of CP and the use of HINE, as well as attitudes, opinions, and perceptions. In April 2022, three consecutive workshops focused on evaluation with HINE were conducted. Three physiotherapists—team leaders for administering HINE in their clinical routines—independently scored the neurological evaluation of 11 children aged between 3 and 12 months. The fourth examiner was a professional with experience with the tool (AH-R).

### 2.6. Statistical Analysis

The required number of individuals was calculated, considering the formulas for calculating the sample size in concordance studies (Zou) [20]. A significance level of 5%, a power of 80%, an expected correlation coefficient of 0.90, and a minimum coefficient of 0.75 were set, for which studies of cross-cultural adaptation and validation of HINE in Indian and Turkish populations were taken into account [11,12]. In addition, a 10% possible loss was considered between valuations. Thus, a sample size of 25 individuals was obtained. 

Statistical analysis was performed with statistical package SPSS v.24 for Windows. Descriptive analysis included means and standard deviations for quantitative variables that followed a normal distribution and a median (Q1–Q3) for those that did not have a normal distribution. Normality was assessed using the Kolmogorov–Smirnov test with the correction of Lilliefors. Reliability analysis was calculated using two approaches. Weighted kappa (κ) analysis was used to determine inter-examiner reliability for the categorical items with 7 response options: 0, 0.5, 1, 1.5, 2, 2.5, and 3. Weighted κ reflects the level of agreement between raters by assigning greater importance to large differences between ratings than to small differences [21]. The linear weighting method was used for calculation. According to Landis and Koch, the strength of agreement of κ is considered slight between 0.00 and 0.20, fair between 0.21 and 0.40, moderate between 0.41 and 0.60, substantial between 0.61 and 0.80, and almost perfect between 0.81 and 1.00 [22].

The ICC (2,1) was obtained using the two-way mixed-effects and absolute agreement model. In all cases, ICC indices were obtained for the total score and asymmetries of the neurological evaluation of HINE and for the different scores that made up the subsections of the neurological evaluation. The confidence interval employed was 95%, therefore values of *p* < 0.05 were considered significant. As reliability criteria, those established by Shrout et al. [23] were used, which state the following: if ICC > 0.80, reliability is considered excellent; if 0.60 < ICC ≤ 0.80, reliability is considered good; if 0.40 < ICC ≤ 0.60, reliability is considered moderate; and if ICC ≤ 0.40, reliability is considered weak or poor.

## 3. Results

### 3.1. Cross-Cultural Translation and Adaptation

After the back-translation process and based on the recommendations of one of the original authors of HINE (LH), four possible comprehension errors were established concerning items of auditory response, hands, quality of movements, and lateral suspension. We proceeded to clarify the back-translated concepts that could lead to comprehension errors (Table 2) to ensure a conceptually equivalent wording in Spanish to the original English version of HINE (Section S1).

### 3.2. Participants

Caregivers of 25 participants between the ages of 3 and 15 months (8.8 months on average) were invited to participate in the intra- and inter-examiner reliability study. Table 3 summarizes the characteristics of the study group.

### 3.3. Evaluation of Intra- and Inter-Examiner Reliability

The results denoted an excellent intra-examiner agreement (MR-M): 0.98 for the total HINE score, and in a range between 0.90 and 0.98 for the subsections (Table 4). Regarding inter-examiner reliability, excellent values were again found for total scores (ICC MR-R, AH-R, JM-A = 0.98). For the subsections, all but three of the ICCs were greater than 0.80, obtaining good reliability for cranial nerve function (ICC AH-R, JM-A = 0.79) and movements (ICC MR-R, JM-A = 0.79) and moderate for asymmetries (range 0.45–0.58).

For individual items, the weighted κ values of inter-examiner agreement between in-person assessment (MR-M) and the video recording (AH-R) ranged from 0.17 to 0.92, with 85% of the items demonstrating at least moderate agreement (κ > 0.41). Substantial agreement (κ = 0.61–0.80) was observed for 10 out of 26 items—including arms, quantity of movements, scarf sign, passive shoulder elevation, pronation/supination, pull to sit, ventral suspension, arm protection, lateral tilting, and forward parachute—while agreement on auditory response and quality of movements were almost perfect (κ > 0.81). Moderate agreement was found for 10 out of 26 items, and fair agreement (κ = 0.21–0.40) for 3 out of 26. The only exception, with slight agreement, was vertical suspension (κ = 0.17) (Table 5). When considering optimal results (scores 2 and 3) together, they constituted 88% (MR-R) and 80% (AH-R) of the results for vertical suspension, revealing that disagreement in that item was found for scores 2 and 3.

### 3.4. Evaluation of Implementation and Inter-Examiner Reliability One Year after the Training Program

Eleven Spanish professionals with multiple specialties—three physiotherapists, three speech therapists, three psychologists, and two occupational therapists—received training in HINE and completed a pre-survey and a survey one year after the training program. They were at the time performing their care work in child development and early intervention services; 54% had less than 5 years of experience in early intervention, 36% between 5 and 15 years, and 9% between 16 and 20 years.

**Figure 1:**
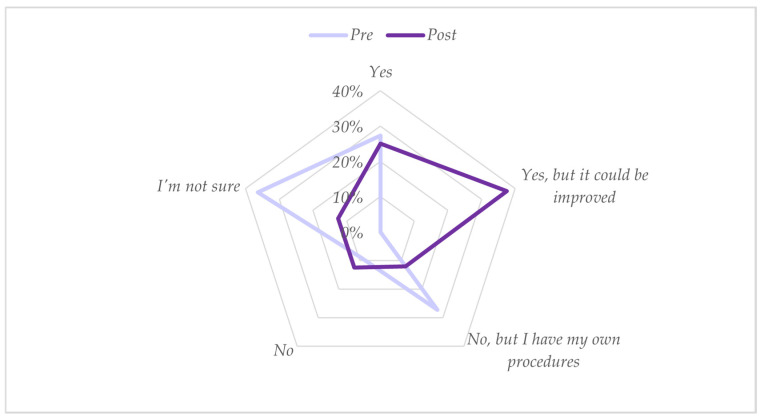
Responses (*n* = 11) to the question “Does your workplace have any standardized protocols, procedures, or reference guidelines for the referral of children at high risk of CP?”

In the block of questions concerning the use of early detection tools in CP, there was a shift in positive responses (“Yes” and “Yes, but it could be improved”) to the question “Does your workplace have any protocol, procedure or standardized reference guidelines for the referral of children at high risk of CP?” The percentage increased from 27% before training to 63% post-training (Figure 1).

In this way, the familiarity with the early detection tools contemplated in the international guidelines also increased. Familiarization with HINE increased by 82%, and with Prechtl’s General Movement Assessment by 91%. For the Alberta Infant Motor Scale and the Bayley Scales of Infant and Toddler Development, familiarization also increased by 9% each, and with the Peabody Developmental Motor Scales-2 by 18%. However, there was no increase in familiarity with the Test of Infant Motor Performance—9% of the participants.

**Figure 2:**
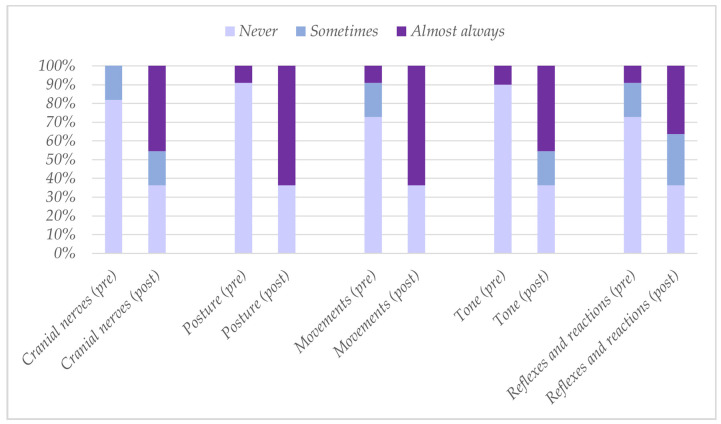
Responses (*n* = 11) to the question “In your clinical practice, how much of the HINE (or isolated HINE items) do you usually record?”

In the block on the use of HINE, the professionals reported recording “sometimes” or “almost always” a higher percentage of HINE or HINE items isolated during their clinical practice, increasing after training for all subsections (Figure 2).

**Figure 3:**
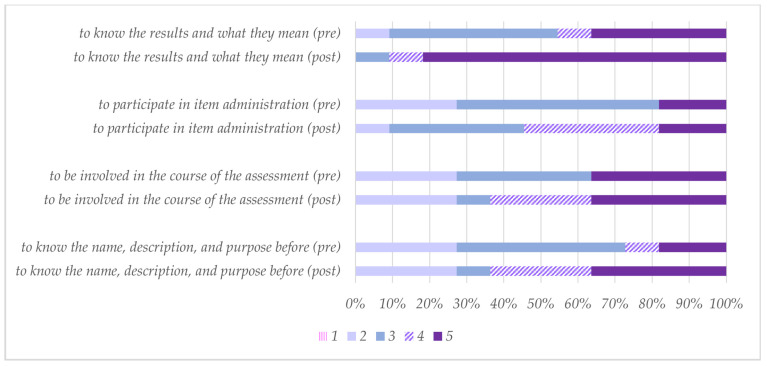
Responses (*n* = 11) to the questions “Families want…” Degree of agreement from 1 (strongly disagree) to 5 (strongly agree).

Some questions referred to attitudes and opinions towards HINE, rating the degree of agreement from 1 (strongly disagree) to 5 (strongly agree). The question “I believe that the HINE is a useful tool to help children and their families” was rated before training at 3 (36%), 4 (36%), and 5 (27%), and after training at 2 (9%) and 5 (91%). Additionally, questions were asked about the opinions of HINE in relation to the families (Figure 3). In this regard, if “medical services/other specialists want to know the results of the HINE and its meaning” the 2 (27%), 3 (64%), and 5 (9%) evolved to 1 (9%), 2 (27%), 3 (9%), 4 (27%), and 5 (27%).

**Figure 4:**
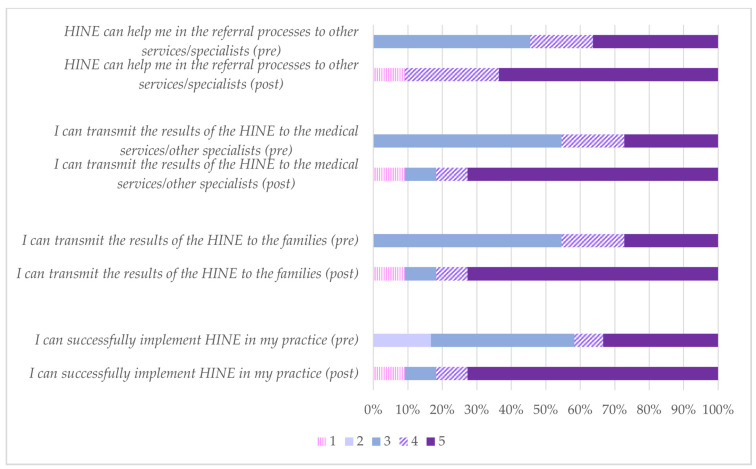
Responses (*n* = 11) to the questions “I trust that…” Degree of agreement from 1 (strongly disagree) to 5 (strongly agree).

Regarding the perception of HINE, the professionals increased their confidence in processes related to the use of HINE for referral to other services or specialists, transmission of results to families and medical services, and successful implementation of the tool in practice (Figure 4).

The inter-examiner reliability of the Spanish version of HINE one year after the training program showed excellent values for the total score (ICC = 0.99), as well as for cranial nerves, posture, tone and reflexes, and reactions (ICCs between 0.86 and 0.98). The movements and the recording of asymmetries showed good reliability (ICC = 0.79 and 0.62, respectively) (Table 6).

## 4. Discussion

This study focuses on the effective implementation of HINE in Spain through its translation and cross-cultural adaptation and the adaptation of a training program, a guideline of recommendations, and adapted materials. Likewise, it includes the study of intra- and inter-examiner reliability of its administration in children at identifiable risk of CP at birth or in the child aged between 3 and 15 months.

The results of the study suggest that the Spanish version of HINE is reliable for application in the Spanish population, with excellent levels of intra-examiner (ICC = 0.98) and inter-examiner reliability (ICC = 0.98). These results contribute to the growing body of research on the psychometric properties of HINE across diverse international contexts. In the original study by Haataja et al. [10], excellent inter-examiner reliability was reported, with an ICC “close to 1” in 25 participants without neurological risk aged 12 and 18 months. Similarly, studies investigating the psychometric properties of HINE in Indian [11], Turkish [12], Italian [14], and Norwegian [15] populations reported comparable inter-examiner reliability values for the total score (ICC = 0.98, 0.97, 0.93, and 0.95, respectively). The excellent intra-examiner reliability observed by Tedla et al. and Adigüzel et al. (ICC = 0.99 and 1.00, respectively) [11,12] is also consistent with the results obtained in this Spanish version for an equivalent population group. A study in the US, focusing on infants born very preterm and using a different method —without video recording analysis but with 26.8 ± 5.16 days between in-person assessments for HINE—resulted in an ICC of 0.79 [13].

In relation to the different subsections, to our knowledge, this is the first study that collects the reliability results for the registration of asymmetries, based on recent recommendations regarding the importance of their registration in the context of evaluation with HINE for the early detection of unilateral CP [24,25]. The good intra-examiner reliability presented by the asymmetries in the Spanish version (ICC = 0.96) contrasts with the moderate values of inter-examiner reliability (ICC = 0.58), which could suggest the need to incorporate descriptors that establish unified criteria for the score of asymmetries in each item of the tool, also highlighting those identified in the literature with a higher correlation with unilateral CP. This would increase the reliability of HINE by reporting asymmetric signs for resistance to passive movement, muscle rigidity, manual reach, or spontaneous kicking, among others [26].

Regarding the rest of the subsections studied in the Indian, Turkish, and Norwegian populations, the excellent inter-examiner reliability for all values (ICC from 0.90 to 1.00, 0.84 to 0.99, and 0.78 to 0.97, respectively) was also obtained in the Spanish version (ICC from 0.87 to 0.94). In this way, a formal training in HINE together with the score sheet—which contains instructions for obtaining the requested answers in each item, illustrative diagrams, and brief descriptions for determining the score according to the degree of response—seems sufficient for its effective implementation in follow-up programs of newborns at risk of CP with professionals trained in HINE and experienced in the management and evaluation of neurodevelopmental disorders. In the absence of a user manual, a guideline of recommendations available in several languages can help improve these training processes.

The evaluation was conducted through video recording by two of the evaluators, following the methodology from other studies on psychometric properties [11,12]. Experiences of the telematic follow-up of children at “high risk” during the COVID-19 pandemic seemed to open a door to the incorporation of HINE within the evaluation processes in telemedicine [27]. This approach could enhance monitoring, facilitate recorded reviews, and potentially prevent delayed diagnosis and referral. Additionally, in the context of implementation and training, video methods enable late review, ensure consistency and reliability, and, if needed, allow revision by other skilled assessors [28].

The inter-examiner reliability for the single items between in-person and video-recorded assessments—not previously reported for HINE—revealed that 85% of the items demonstrated at least moderate agreement. Possible limitations for the evaluation of certain items of tone and/or reflexes and reactions, due to their subjective impact [29] and the lack of hands-on management of children, do not seem to influence the examiner’s perception. This is illustrated by the intra- and inter-examiner reliability obtained for both subsections.

Interestingly, 11 out of 12 (92%) of the HINE items shown to be more predictive for CP [30] had at least moderate agreement in this study. Seven of them showed substantial agreement (quantity of movements, scarf sign, pronation/supination, ventral suspension, arm protection, lateral tilting, and forward parachute), and quality of movements demonstrated “almost perfect” κ strength of agreement. This suggests promise for a short screening version of HINE that could be video-recorded. Similarly, exploring an adapted version of the tool allowing family participation in requesting items could follow examples set by Prechtl’s General Movement Assessment [31], the Alberta Infant Motor Scale [32], or the Gross Motor Function Measure [33].

HINE has additional advantages compared with other neurological evaluations. It requires a shorter administration time—it can be completed in 5 to 15 min [8,17]—than the Amiel–Tison Neurologic Assessment [34] or the Touwen Infant Neurological Examination [35], which are more extensive and present some items that are difficult to obtain in a routine clinical environment. It is also described as a neurological evaluation that can be administered even by students or professionals with limited experience [7,8,36,37,38]. This would differentiate it from other neurological evaluations that require examiners with extensive experience, lengthy certification processes, significant costs to acquire the evaluation, and/or considerable time for its administration. These benefits seem to be directed specifically to the main barriers identified by professionals, such as Spanish physiotherapists, concerning the use of early detection tools for CP [39].

This study has investigated the impact of standardized training, through the modules proposed by Maitre et al. [7], in professionals with one year of experience with the tool. Excellent reliability values were obtained for the Spanish version, in addition to increasing the incorporation of the HINE items in clinical practice—in accordance with Maitre et al. [7]—but also the sensitization of professionals regarding the need for the existence and improvement of referral protocols for children at high risk of CP. One year after the training, the professionals’ perception about what “families want” seemed much more aligned with the reality expressed by them in qualitative studies [40]. As a result, awareness was raised about the transmission of the name, description, and purpose of HINE, as well as the results obtained and their significance. In addition, the importance of parental involvement in the course of the evaluation was strengthened, even their participation in the administration of items. In parallel, the professionals’ confidence in carrying out these processes increased substantially.

Scientific evidence supports HINE’s ability to predict CP [4] and distinguish between unilateral and bilateral CP, as well as the prognosis of the motor function [41]. This aspect needs further exploration for the Spanish version, representing a limitation of this feasibility project. The roles of various healthcare professions —including neonatologists, developmental pediatricians, and/or neurologists [42]—as crucial figures in the neurological examination, should also be investigated. Additionally, based on the available evidence, the use of the Spanish version of HINE should be promoted together with Prechtl’s General Movement Assessment and other motor assessment tools (such as the Infant Motor Profile test and the Alberta Infant Motor Scale) for the description of the population at “high risk” of development of CP [6] and the follow-up population with identifiable risk at birth or in the child, in Spain [43]. The Spanish from Spain version of the HINE proforma could likewise serve for further cross-cultural adaptations to other Spanish-speaking countries in Latin America.

## 5. Conclusions

The results of the intra- and inter-examiner reliability study of the Spanish version of HINE indicate that it may be a reliable measure for the neurological evaluation of children at risk of CP between 3 and 15 months of age. It can be administered after a standardized training of 4 h, evaluated through video recording without costs to acquire the evaluation. This would allow its accessible and generalized implementation in the clinical context. Future studies and implementation procedures are needed to improve the assessment of asymmetries within HINE as an important component in supporting the processes of early detection of unilateral CP.

## Figures and Tables

**Table 1:** HINE training modules. Adapted from Maitre et al. [6].

Training Elements	Duration
Reading the scientific literature supporting the use of HINE	30 min
Review of the administration and scoring of individual HINE items	45 min
HINE demonstrations with video recordings and face-to-face assessments (different ages and health statuses)	15 min per patient
Review of video recordings and discussion of HINE administration and scoring	20 min per patient

**Table 2:** Clarification of back-translated concepts with possible misunderstandings.

Item	Subsection	Possible Comprehension Errors
Auditory response	Cranial nerves	The Spanish translation should clarify the concept of “dubious reaction” to the stimulus.
Hands	Posture	In the Spanish translation, there should be the concept of “fisting”.
Quality	Movements	In the Spanish translation, there should be a word conceptually equivalent to “spasmodic”.
Lateral suspension	Reflexes and reactions	The Spanish translation should make it clear that the movement is “towards” the horizontal, not in the full horizontal position.

**Table 3:** Characteristics of the participants.

Characteristic	Total Study Population (*n* = 25)
Gestational age mean (SD) (minimum, maximum), wks	30.3 (4.5) (24.4–41.0)
Birth weight mean (SD) (minimum, maximum), g	1512.6 (741.1) (720–3450)
Head circumference, mean (SD) (minimum, maximum), cm	27.8 (4.1) (21.5–35)
Apgar 1′ mean (SD) (minimum, maximum); Apgar 1′ mean (SD) (minimum, maximum)	6.5 (1.9) (2–9); 8.1 (1.6) (4–10)
Multiple birth, *n* (%)	8 (32)
Preterm birth (<37 wks gestation), *n* (%)	23 (92)
HIE, *n* (%)	1 (4)
BPD, *n* (%)	2 (8)
Major brain pathologies in neuroimaging ^a^, *n* (%)	3 (12)
Age at HINE assessment mean (SD) (minimum, maximum), mo	8.8 (3.0) (3.5–15.0)
3 to 5 mo, *n* (%)	4 (16)
6 to 8 mo, *n* (%)	9 (36)
9 to 15 mo, *n* (%)	12 (48)

^a^ Neuroimaging (ultrasound, magnetic resonance imaging) was performed routinely in the neonatal intensive care unit; BPD—bronchopulmonary dysplasia; HIE—hypoxic-ischemic encephalopathy.

**Table 4:** Intra- and inter-examiner reliability of the Spanish version of HINE.

Intra-Examiner Reliability	Cranial Nerves	Posture	Movements	Tone	Reflexes and Reactions	Asymmetries	Total Score
MR-M	0.904	0.955	0.980	0.918	0.972	0.959	0.981
	<0.001	<0.001	<0.001	<0.001	<0.001	<0.001	<0.001
**Inter-Examiner Reliability**							
MR-R, AH-R	0.817	0.818	0.990	0.868	0.914	0.507	0.968
	<0.001	<0.001	<0.001	<0.001	<0.001	0.003	<0.001
MR-R, JM-A	0.854	0.868	0.794	0.970	0.888	0.451	0.975
	<0.001	<0.001	<0.001	<0.001	<0.001	0.050	<0.001
AH-R, JM-A	0.786	0.832	0.816	0.868	0.867	0.489	0.976
	<0.001	<0.001	<0.001	<0.001	<0.001	0.037	<0.001
MR-R, AH-R, JM-A	0.870	0.887	0.903	0.936	0.924	0.583	0.982
	<0.001	<0.001	<0.001	<0.001	<0.001	0.001	<0.001

The higher values indicate the ICCs; the lower values are the corresponding *p*-values.

**Table 5:** Inter-examiner reliability of single HINE items.

**Inter-Examiner Reliability** MR-R, AH-R	***Cranial nerve function***	**Facial appearance**	**Eye movements**	**Visual response**	**Auditory response**	**Sucking/swallowing**			
		0.359	0.468	0.490	0.917	0.370			
		0.022	0.001	0.004	<0.001	0.006			
	***Posture***	**Head**	**Trunk**	**Arms**	**Hands**	**Legs**	**Feet**		
		0.419	0.596	0.648	0.259	0.545	0.527		
		0.006	<0.001	0.001	0.080	<0.001	<0.001		
	***Movements***	**Quantity**	**Quality**						
		0.684	0.855						
		<0.001	<0.001						
	***Tone***	**Scarf sign**	**Passive shoulder elevation**	**Pronation/supination**	**Hip adductors**	**Popliteal angle**	**Ankle dorsiflexion**	**Pull to sit**	**Ventral suspension**
		0.658	0.648	0.648	0.468	0.552	0.416	0.779	0.795
		<0.001	0.001	0.001	0.006	0.001	0.007	<0.001	<0.001
	***Reflexes and reactions***	**Arm protection**	**Vertical suspension**	**Lateral tilting**	**Forward parachute**	**Tendon reflexes**			
		0.627	0.172	0.663	0.631	0.479			
		<0.001	0.380	<0.001	<0.001	0.001			

The higher values indicate the weighted κ; the lower values are the corresponding *p*-values.

**Table 6:** Inter-examiner reliability of the Spanish version of HINE one year after the training program.

Inter-Examiner Reliability	Cranial Nerves	Posture	Movements	Tone	Reflexes and Reactions	Asymmetries	Total Score
AH-R, IA-G, CB-M, AMF-T	0.945	0.973	0.790	0.856	0.980	0.618	0.988
Total score	<0.001	<0.001	<0.001	<0.001	<0.001	0.026	<0.001

The higher values indicate the ICCs; the lower values are the corresponding *p*-values.

## Data Availability

Data are contained within the article or Supplementary Materials.

## Supplementary Materials

The following supporting information can be downloaded at: https://www.mdpi.com/article/10.3390/healthcare12030380/s1, Section S1: HINE proforma in Spanish.
