# Supplementary material for: Reliability, Knowledge Translation, and Implementability of the Spanish Version of the Hammersmith Infant Neurological Examination
Source: Healthcare (Basel). 2024 Feb 1;12(3):380. doi: 10.3390/healthcare12030380 (PMC10855046; doi:10.3390/healthcare12030380)
Supplement: Supplementary file 1 [file healthcare-12-00380-s001.zip › healthcare-2783644-supplementary.pdf]

HAMMERSMITH INFANT NEUROLOGICAL EXAMINATION (v 07.07.17)

Name **Date of birth**

Gestational age **Date of examination**

Chronological age / Corrected age **Head circumference**

SUMMARY OF EXAMINATION
**Global score (max 78)**
**Number of asymmetries**
**Behavioural score (not part of the optimality score)**

Cranial nerve function score (max 15)
Posture score (max 18)
Movements score (max 6)
Tone score (max 24)
Reflexes and reactions score (max 15)

COMMENTS

(Throughout the exam, if a response is not optimal but not poor enough to score 1, give a score of 2)

NEUROLOGICAL EXAMINATION

ASSESSMENT OF CRANIAL NERVE FUNCTION

	score 3	2	score 1	score 0	score	Asymmetry / Comments
**Facial appearance** (at rest and when crying or stimulated)	Smiles or reacts to stimuli by closing eyes and grimacing		Closes eyes but not tightly, poor facial expression	Expressionless, does not react to stimuli		
**Eye movements**	Normal conjugate eye movements		**Intermittent** Deviation of eyes or abnormal movements	**Continuous** Deviation of eyes or abnormal movements		
**Visual response** Test ability to follow a black/white target	Follows the target in a complete arc		Follows target in an incomplete or asymmetrical arc	Does not follow the target		
**Auditory response** Test the response to a rattle	Reacts to stimuli from both sides		Doubtful reaction to stimuli or asymmetry of response	No response		
**Sucking/swallowing** Watch infant suck on breast or bottle. If older, ask about feeding, assoc. cough, excessive dribbling	Good suck and swallowing		Poor suck and/or swallow	No sucking reflex, no swallowing		

ASSESSMENT OF POSTURE (note any asymmetries)

	score 3	score 2	score 1	score 0	sc	Asymmetry / comments
**Head**<br>in sitting	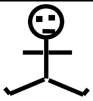<br>Straight; in midline		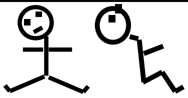<br>Slightly to side *or* backward *or* forward	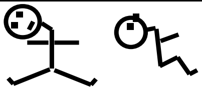<br>Markedly to side *or* backward *or* forward		
**Trunk**<br>in sitting	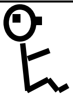<br>Straight		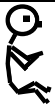<br>Slightly curved or bent to side	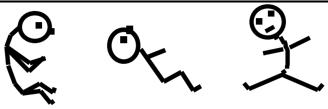<br>Very rounded / rocketing back / bent sideway		
**Arms**<br>at rest	In a neutral position, central straight or slightly bent		**Slight** internal rotation *or* external rotation<br><br>**Intermittent** dystonic posture	**Marked** internal rotation *or* external rotation or<br><br>dystonic posture hemiplegic posture		
**Hands**	Hands open		**Intermittent** adducted thumb *or* fisting	**Persistent** adducted thumb *or* fisting		
**Legs**<br>in sitting	Able to sit with a straight back and legs straight or slightly bent (long sitting)<br>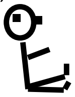		Sit with straight back but knees bent at 15-20 °<br>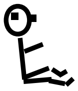	Unable to sit straight unless knees markedly bent (no long sitting)<br>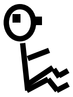		
in supine and in standing	Legs in neutral position straight *or* slightly bent	**Slight** internal rotation or external rotation	Internal rotation *or* external rotation at the hips	**Marked** internal rotation *or* external rotation *or* fixed extension or flexion or contractures at hips and knees		
**Feet**<br>in supine and in standing	Central in neutral position<br><br>Toes straight midway between flexion and extension		**Slight** internal rotation *or* external rotation<br><br>**Intermittent** Tendency to stand on tiptoes or toes up or curling under	**Marked** internal rotation *or* external rotation at the ankle<br><br>**Persistent** Tendency to stand on tiptoes *or* toes up or curling under		

ASSESSMENT OF MOVEMENTS

	Score 3	Score 2	Score 1	Score 0	score	Asymmetry / comments
**Quantity**<br>Watch infant lying in supine	Normal		Excessive or sluggish	Minimal or none		
**Quality**<br>Observe infant's spontaneous voluntary motor activity during the course of the assessment	Free, alternating, and smooth		Jerky<br><br>Slight tremor	• Cramped & synchronous<br>• Extensor spasms<br>• Athetoid<br>• Ataxic<br>• Very tremulous<br>• Myoclonic spasm<br>• Dystonic movement		

ASSESSMENT OF TONE

	Score 3	Score 2	Score 1	Score 0	sc	Asym/Co
**Scarf sign** Take the infant's hand and pull the arm across the chest until there is resistance. Note the position of the elbow in relation to the midline.	**Range:** 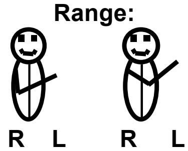 **R L R L**		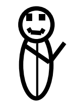 **R L**	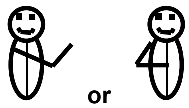 **R L** or **R L**		
**Passive shoulder elevation** Lift arm up alongside infant's head. Note resistance at shoulder and elbow.	Resistance overcomeable 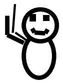 **R L**	Resistance difficult to overcome **R L**	No resistance 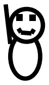 **R L**	Resistance, not overcomeable 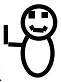 **R L**		
**Pronation/supination** Steady the upper arm while pronating and supinating forearm, note resistance	Full pronation and supination, no resistance		Resistance to full pronation / supination overcomeable	Full pronation and supination not possible, marked resistance		
**Hip adductors** With both the infant's legs extended, abduct them as far as possible. The angle formed by the legs is noted.	**Range: 150-80°** 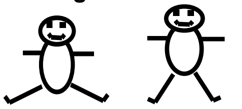 **R L R L**	**150-160°** 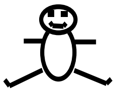 **R L**	**>170°** 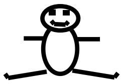 **R L**	**<80°** 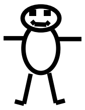 **R L**		
**Popliteal angle** Keeping the infant's bottom on the bed, flex both hips onto the abdomen, then extend the knees until there is resistance. Note the angle between upper and lower leg.	**Range: 150°-100°** 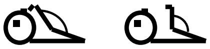 **R L R L**	**150-160°** 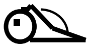 **R L**	**~90° or > 170°** 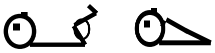 **R L R L**	**<80°** 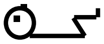 **R L**		
**Ankle dorsiflexion** With knee extended, dorsiflex the ankle. Note the angle between foot and leg.	**Range: 30°-85°** 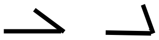 **R L R L**	**20-30°** 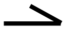 **R L**	**<20°or 90°** 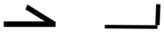 **R L R L**	**> 90°** 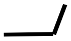 **R L**		
**Pull to sit** Pull infant to sit by the wrists. (support head if necessary)	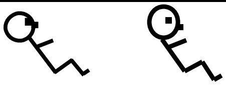		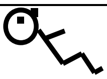	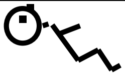		
**Ventral suspension** Hold infant horizontally around trunk in ventral suspension; note position of back, limbs and head.	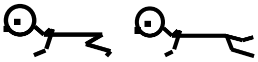		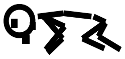	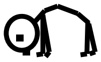		

REFLEXES AND REACTIONS

	Score 3	Score 2	Score 1	Score 0	sc	Asym / Co
**Arm protection** Pull the infant by one arm from the supine position (steady the contralateral hip) and note the reaction of arm on opposite side.	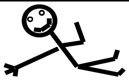 Arm & hand extend **R L**		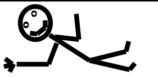 Arm semi-flexed **R L**	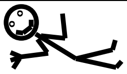 Arm fully flexed **R L**		
**Vertical suspension** hold infant under axilla making sure legs do not touch any surface – you may "tickle" feet to stimulate kicking.	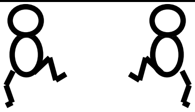 Kicks symmetrically		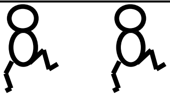 Kicks one leg more or poor kicking	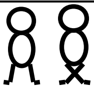 No kicking even if stimulated or scissoring		
**Lateral tilting** (describe side up**)**. Hold infant up vertically near to hips and tilt sideways towards the horizontal. Note response of trunk, spine, limbs and head.	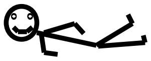 **R L**	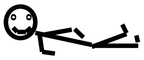 **R L**	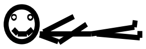 **R L**	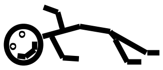 **R L**		
**Forward parachute** Hold infant up vertically and quickly tilt forwards. Note reaction /symmetry of arm responses,	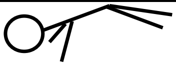 (after 6 months)		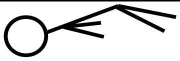 (after 6 months)			
**Tendon Reflexes** Have child relaxed, sitting or lying – use small hammer	Easily elicitable biceps knee ankle	Mildly brisk bicep knee ankle	Brisk biceps knee ankle	Clonus or absent biceps knee ankle		

SECTION 2 MOTOR MILESTONES (not scored; note asymmetries)

**Head control**	Unable to maintain head upright<br>normal to 3m	Wobbles<br>normal up to 4m	Maintained upright all the time<br>normal from 5m			**Please note age at which maximum skill is achieved**
**Sitting**	Cannot sit	With support at hips 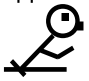<br>normal at 4m	Props 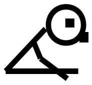<br>normal at 6m	Stable sit 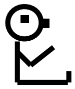<br>normal at 7-8m	Pivots (rotates) 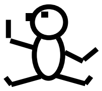<br>normal at 9m	Observed:<br>Reported (age):
**Voluntary grasp – note side**	No grasp	Uses whole hand	Index finger and thumb but immature grasp	Pincer grasp		Observed:<br>Reported (age):
**Ability to kick in supine**	No kicking	Kicks horizontally but legs do not lift	Upward (vertically) 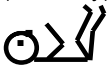<br>normal at 3m	Touches leg 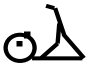<br>normal at 4-5m	Touches toes 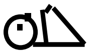<br>normal at 5-6m	Observed:<br>Reported (age):
**Rolling - note through which side(s)**	No rolling	Rolling to side<br>normal at 4m	Prone to supine<br>normal at 6 m	Supine to prone<br>normal at 6 m		Observed:<br>Reported (age):
**Crawling - note if bottom shuffling**	Does not lift head	On elbows 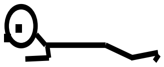<br>normal at 3m	On outstretched hands 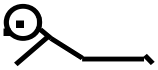<br>normal at 4m	Crawling flat on abdomen 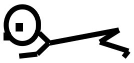<br>normal at 8m	Crawling on hands and knees 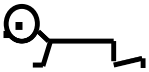<br>normal at 10m	Observed:<br>Reported (age):
**Standing**	Does not support weight	Supports weight<br>normal at 4m	Stands with support<br>normal at 7m	Stands unaided<br>normal at 12m		Observed:<br>Reported (age):
**Walking**		Bouncing<br>normal at 6m	Cruising (walks holding on)<br>normal at 12m	Walking independently<br>normal by 15m		Observed:<br>Reported (age):

SECTION 3 BEHAVIOUR (not scored)

	1	2	3	4	5	6	Comment
**Conscious state**	Unrousable	Drowsy	Sleep but wakes easily	Awake but no interest	Loses interest	Maintains interest	
**Emotional state**	Irritable, not consolable	Irritable, carer can console	Irritable when approached	Neither happy or unhappy	Happy and smiling		
**Social orientation**	Avoiding, withdrawn	Hesitant	Accepts approach	Friendly			

For enquiries about the Hammersmith Infant Neurological examination, please contact either Prof Frances Cowan f.cowan@imperial.ac.uk, Prof Leena Haataja leena.haataja@hus.fi or Prof Eugenio Mercuri eumercuri@gmail.com
